# Manual vs. Automatic Segmentation in Infrared Thermography: Inter-Rater Reliability and a Longitudinal Single-Case Feasibility Illustration of Agreement for Musculoskeletal Rehabilitation Monitoring

**DOI:** 10.3390/jfmk11030285

**Published:** 2026-07-22

**Authors:** Lorenzo Accurso, Vincent Couturier, Tristan Castonguay, Julie Lamoureux, Geoffrey Dover

**Affiliations:** 1 Department of Health, Kinesiology, and Applied Physiology, Concordia University, Montreal, QC H4B 1R6, Canada; lorenzoaccurso@hotmail.com (L.A.); vincent.couturier@hotmail.com (V.C.); tristan.castonguay@concordia.ca (T.C.); julie.lamoureuxx@gmail.com (J.L.); 2 Centre for Interdisciplinary Research in Rehabilitation of Greater Montreal (CRIR), Montreal, QC H7V 1R2, Canada; 3 School of Health, Concordia University, Montreal, QC H4B 1R6, Canada

**Keywords:** infrared thermography, thermal imaging, skin temperature, injury monitoring, rehabilitation, software comparison, inter-rater reliability, agreement, FLIR Thermal Studio Pro™, ThermoHuman^®^

## Abstract

**Background**: Infrared thermography (IRT) offers a non-invasive means to monitor skin temperature and physiological changes during rehabilitation. However, variability in image segmentation methods limits its broader clinical use. This study assessed the inter-rater reliability of a manual thermal image segmenatation protocol using FLIR Thermal Studio Pro™ and presents a longitudinal single-case feasibility illustration evaluating the agreement between this manual method and automatic segmentation using ThermoHuman^®^. **Methods**: Two regions of interest (ROI) were analyzed throughout the study: the frontal knee and vastus medialis oblique (VMO). For the inter-rater reliability analysis, thermal images from 20 professional athletes were used. For the agreement analysis, 50 lower extremity thermal images were captured from a single athlete recovering from a meniscal repair over a four-month rehabilitation period. Intraclass correlation coefficients (ICC _(2,1)_) were used to assess agreement and reliability. **Results**: Inter-rater reliability was excellent for all frontal knee measurements (ICC _(2,1)_ = 0.991, 0.995, and 0.964 for maximum, average, and minimum temperature, respectively) and for VMO maximum and average temperature (ICC _(2,1)_ = 0.967 and 0.981, respectively), with VMO minimum temperature as the exception demonstrating good reliability (ICC _(2,1)_ = 0.779). Excellent agreement was found between methods for all frontal knee (ICC _(2,1)_ = 0.967, 0.949, and 0.985) and VMO measurements (ICC _(2,1)_ = 0.971, 0.973, and 0.980) for maximum, average, and minimum temperature, respectively. **Conclusion**: Manual segmentation using FLIR Thermal Studio Pro™ provides highly reliable results across multiple subjects, supporting its use as a practical and consistent clinical tool. Furthermore, our single-case feasibility illustration suggests it provides comparable results to automatic segmentation using ThermoHuman^®^, supporting its potential feasibility for accessible rehabilitation monitoring in injured athletes. Further multi-subject research is warranted to confirm the broader applicability of the agreement findings.

## 1. Introduction

Infrared thermography (IRT) is a non-invasive imaging technique that measures skin surface temperature, gaining clinical relevance in musculoskeletal rehabilitation [1,2,3,4]. A key advantage of IRT is its ability to capture consecutive thermal images over time, enabling frequent, real-time monitoring of physiological changes [5,6]. Consequently, IRT serves as a practical alternative to more time-consuming and costly imaging modalities, such as magnetic resonance imaging (MRI), for tracking recovery [7,8,9]. While MRI remains crucial for initial diagnosis and assessing structural integrity, its utility for frequent, longitudinal monitoring during rehabilitation is often limited due to factors such as cost, accessibility, and the persistence of anatomical changes that may not correlate with functional recovery [10,11]. For instance, studies have shown that repeated MRIs do not reliably predict recovery or reinjury risk in athletes with acute hamstring strains, emphasizing the importance of clinical evaluations and functional assessments in guiding return-to-play decisions [10]. Similarly, anatomical changes observed on MRI can outlast functional deficits, highlighting that clinical and functional assessments are often more informative for tracking rehabilitation progress [11]. In this context, IRT offers clear advantages for frequent, low-cost serial assessment, serving as a valuable complementary tool rather than a direct replacement for MRI in rehabilitation monitoring.

To support broader clinical adoption of IRT, consistent and standardized image analysis methods must be established. Software tools interpret thermal images by calculating and visualizing temperature distributions across regions of interest (ROIs). Among the available tools, ThermoHuman^®^ (PEMA THERMO GROUP SL, April 2024 version, Madrid, Spain) is widely used in research for its ability to automatically segment the body into anatomical regions and provide detailed temperature data [12]. However, the per-image processing fee associated with ThermoHuman^®^ can be costly for clinicians or researchers analyzing large datasets. As a more accessible alternative, FLIR Thermal Studio Pro™ (Teledyne FLIR LLC, version 1.9.28, Wilsonville, Oregon, USA) is bundled with the purchase of the FLIR camera and enables manual image segmentation where users manually delineate the ROIs to obtain temperature readings. While this method requires greater anatomical knowledge and operator skill, it provides flexibility in resource-limited clinical environments, as it does not require paying for an additional software subscription. If manual segmentation can be shown to produce results comparable to those of automatic software, it would represent a practical and accessible tool for routine clinical use. Demonstrating strong agreement and inter-rater reliability between manual and automatic thermal image segmentation is therefore critical. Without such agreement and reliability, temperature differences may indicate measurement inconsistency rather than true physiological changes, limiting IRT’s clinical utility for tracking recovery and guiding treatment [13].

While multiple studies have evaluated the reliability of IRT, most have focused on specific conditions or imaging protocols [14,15,16,17,18,19,20]. For instance, high inter-examiner reliability (ICC = 0.865) was reported in patients with complex regional pain syndrome when ROIs were selected based on clinical history and symptoms [14]. Another study on chronic migraine patients found frontal thermography to be reproducible (ICC > 0.75) [15]. A study comparing ThermoHuman^®^ to manual methods for plantar skin temperature assessment found excellent reliability (ICC > 0.8) but was limited to a single anatomical region at two time points [19]. Another investigation tested the reproducibility of temperature changes in the patellar tendon but was confined to healthy athletes [20]. Evaluating temperature changes in an injured athlete is vital for supporting IRT’s clinical adoption. To date, no study has directly compared ThermoHuman^®^ and FLIR Thermal Studio Pro™ for analyzing temperature in an injured knee joint or assessed their interchangeable use across multiple time points during recovery.

This study had two primary objectives: (1) to assess the inter-rater reliability of the manual segmentation protocol across a multi-subject cohort and (2) to evaluate, through a longitudinal single-case feasibility illustration, the agreement between manual thermal image segmentation using FLIR Thermal Studio Pro™ and automatic segmentation using ThermoHuman^®^. We hypothesized that the manual segmentation protocol would show high inter-rater reliability between independent raters and that both segmentation methods would demonstrate excellent agreement.

## 2. Materials and Methods

### 2.1. FLIR E8 Thermal Camera

We used the FLIR E8 Wi-Fi thermal camera (Teledyne FLIR LLC, Wilsonville, OR, USA) to capture all thermal images. This camera was selected because it meets ThermoHuman’s recommended resolution requirements, and its accessibility makes it suitable for both research and clinical use [21,22]. The camera features a 320 × 240-pixel infrared resolution, a 45-degree field of view, a focus-free FOL7 lens, and a thermal sensitivity of <0.15 °C, ensuring accurate detection of subtle temperature variations [23]. The temperature measurement range spans from −20.0 °C to 250.0 °C, and images are captured at a 9 Hz frequency [23]. The camera performs an automatic self-calibration (non-uniformity correction) upon startup and every two minutes thereafter, ensuring accurate temperature readings throughout each imaging session [23].

### 2.2. Thermal Image Acquisition

Thermal imaging was conducted in accordance with the Glamorgan Protocol [24], the Thermographic Imaging in Sports and Exercise Medicine (TISEM) checklist [25], and the American Academy of Thermology (AAT) guidelines [26] to ensure standardized and reproducible image capture. Protocol approaches to defining ROIs and controlling for environmental variables have been recognized and applied in various studies, including some that have similarly monitored physiological responses in clinical settings [27,28].

#### 2.2.1. Equipment and Setup

All imaging sessions were conducted in an environmentally controlled room, with ambient temperature maintained between 20 and 25 °C and relative humidity between 40 and 60%, as recommended by the established protocols [24,25,26]. To minimize radiant interference and background reflections, room lights were turned off, and a non-reflective black backdrop was used.

#### 2.2.2. Image Capture

**Figure 1:**
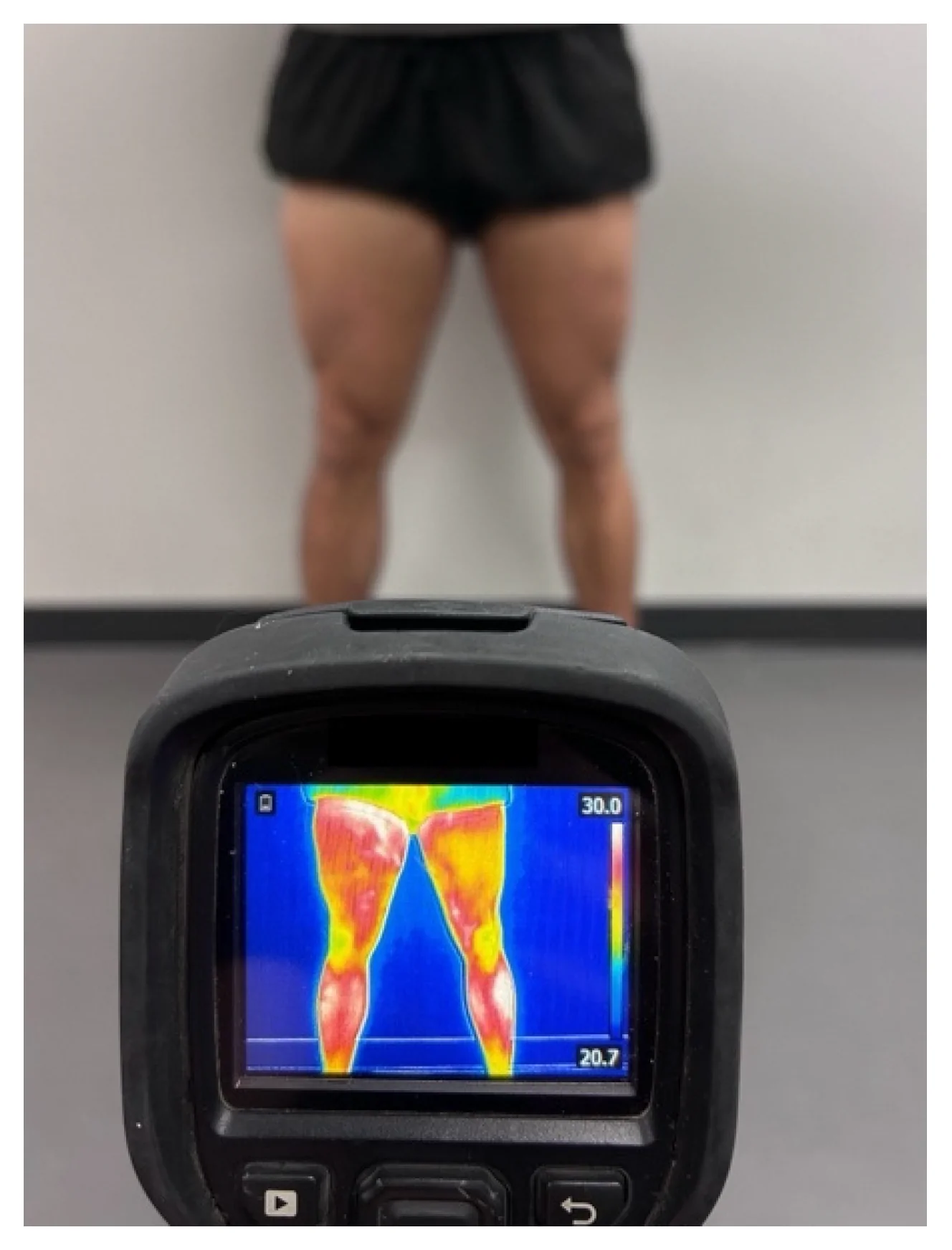
Standardized imaging setup showing the participant positioned five feet from the FLIR E8 Wi-Fi thermal camera, with tape markings on the floor to ensure consistent positioning across sessions. The anterior view spans from the upper thigh to the ankles.

Consistent with the Glamorgan Protocol, the patient was positioned standing five feet from the camera, with the camera lens aligned perpendicularly to the target area to mitigate angular distortions. Floor markings were used to ensure consistent patient positioning across all sessions. The patient’s legs were exposed to allow for clear thermal readings of the anterior aspect of the upper thigh to the mid-shin, which encompassed the two ROIs: the frontal knee and the VMO muscle (Figure 1).

### 2.3. Thermal Image Segmentation

#### 2.3.1. Manual Segmentation Using Thermal Studio Pro™

**Figure 2:**
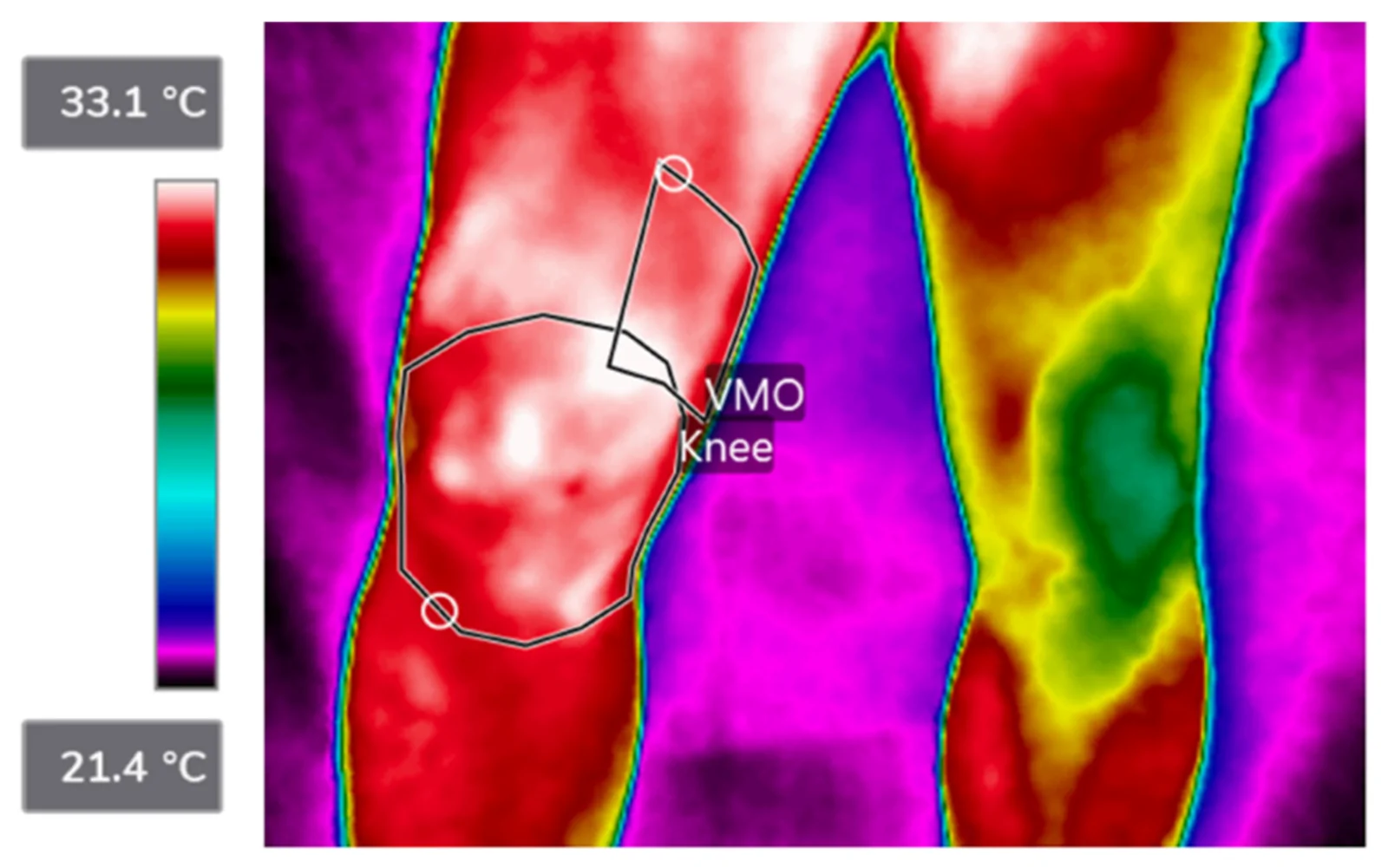
Example of manual segmentation using FLIR Thermal Studio Pro™, showing the delineated ROIs for the frontal knee and VMO muscle of the right leg.

Manual segmentation was performed using FLIR Thermal Studio Pro™. Unlike automatic software, such as ThermoHuman^®^, this tool requires the user to manually delineate ROIs to extract temperature data. A standardized, landmark-based approach was established to ensure reproducibility. An example of the resulting manual segmentation for both ROIs is shown in Figure 2. After segmentation was completed, FLIR Thermal Studio Pro™ calculated the average, maximum, and minimum temperatures for each region.

For the frontal knee, a freehand polygon was drawn with the inferior border anchored at the tibial tuberosity; the superior border was set at the distal end of the quadriceps just superior to the patella; and the medial/lateral borders followed the natural contour of the knee joint. Peripheral skin edges were deliberately excluded to avoid artificially low minimum temperature values.

Similar to the frontal knee, a freehand polygon was drawn for the VMO using identifiable anatomical landmarks. The inferior border was set at the superior pole of the patella, while the medial border followed the inner thigh contour, carefully avoiding the cooler peripheral skin edge. The lateral border was defined at the medial third of the thigh, providing a consistent landmark to delineate the VMO from the adjacent rectus femoris and vastus intermedius musculature. The superior border was drawn approximately 5 cm proximal to the inferior border without extending into more proximal, vertically oriented fibers of the vastus medialis.

#### 2.3.2. Automatic Segmentation Using ThermoHuman^®^

**Figure 3:**
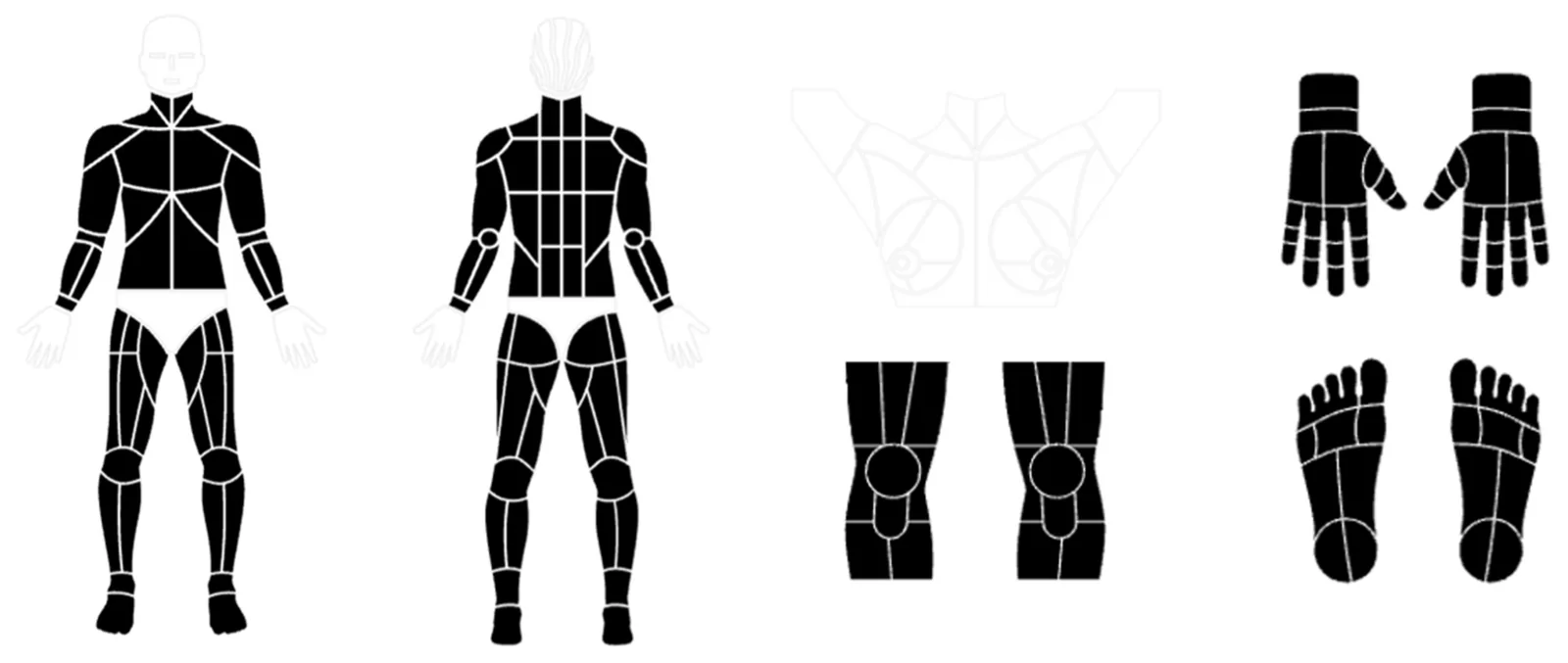
ThermoHuman^®^ interface displaying the ROIs available for automatic segmentation. The software automatically segments the human body into different ROIs and calculates the average, maximum, and minimum temperatures for each.

**Figure 4:**
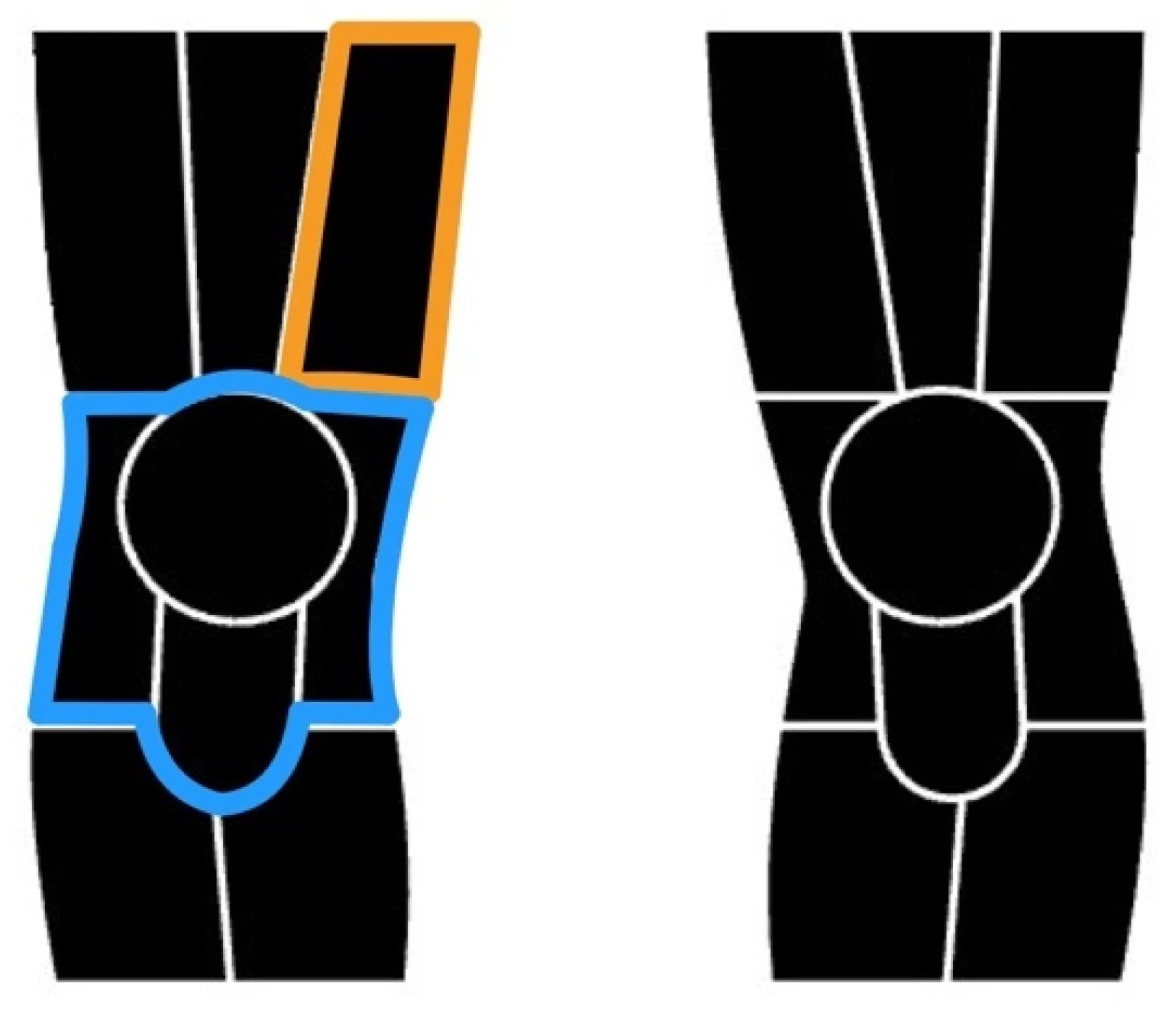
Automatic segmentation of the lower limb using ThermoHuman^®^. The frontal knee ROI (outlined in blue) was a composite of four sub-regions. The VMO ROI (outlined in orange) corresponded to the software’s lower vastus medialis segment.

**Figure 5:**
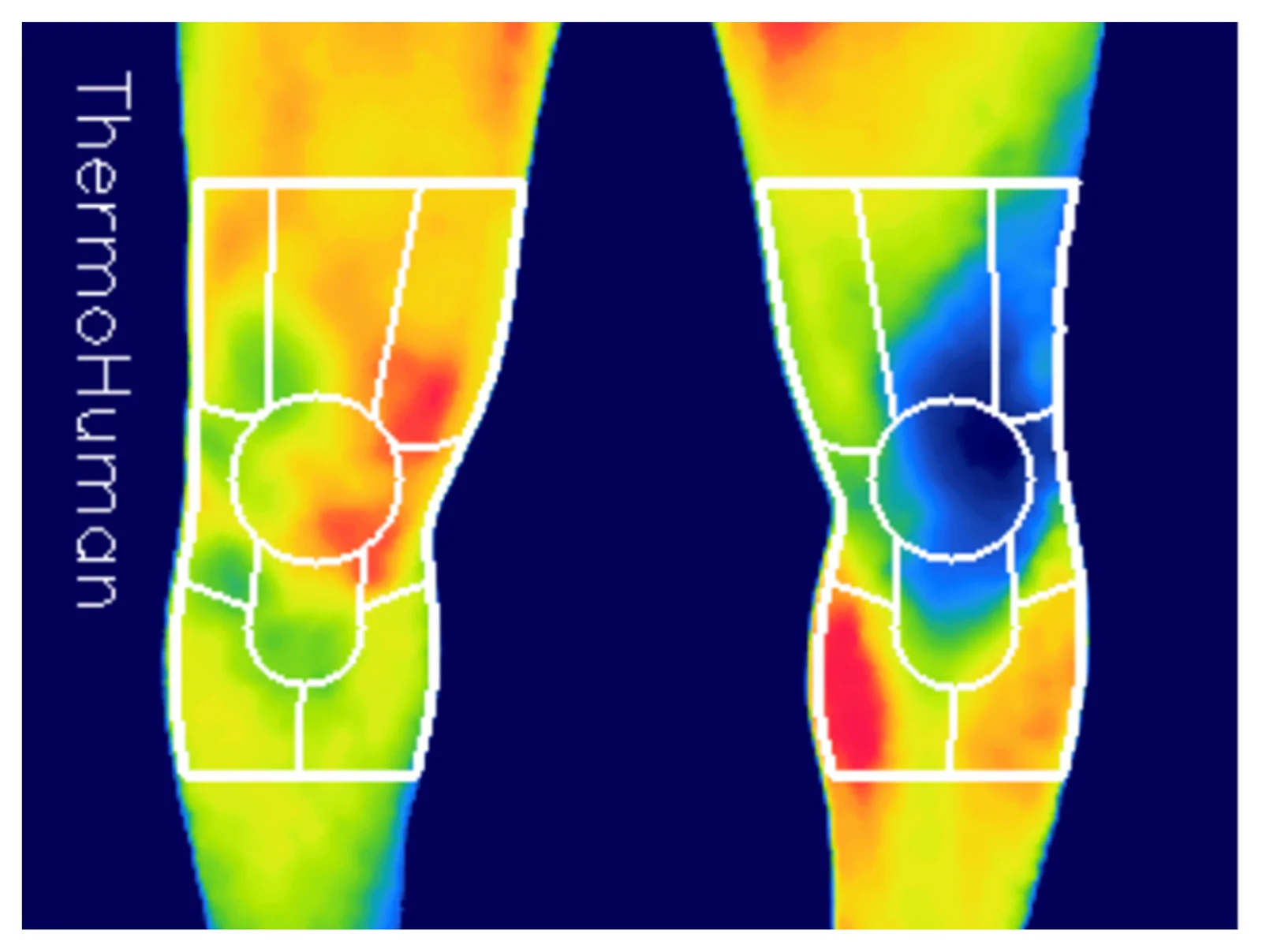
Complete automatic segmentation applied to a thermal image of the leg using ThermoHuman^®^, focusing on areas of the frontal knee, lower thigh, and upper calf.

ThermoHuman^®^ is a software program that automatically segments thermal images into predefined anatomical regions and extracts temperature data for each region (Figure 3). Upon uploading an image, the software automatically identifies ROIs and calculates the average, maximum, and minimum temperatures for each. For this study, we used the knee-specific segmentation option. For the frontal knee, ThermoHuman^®^ separated the outer and inner parts of the frontal knee, the patella, and the patellar tendon (Figure 4). In the lower thigh, attention was given to the lower sections of the vastus medialis, vastus lateralis, and rectus femoris. As shown in Figure 4, we averaged the temperature readings across the four frontal knee regions to produce a single composite value for each metric (average, maximum, and minimum temperature) to ensure a consistent basis for comparison between automatic and manual methods. Figure 5 shows the complete automatic segmentation applied across the full lower limb.

### 2.4. Inter-Rater Reliability of Manual Segmentation

To evaluate the reproducibility of the manual segmentation protocol, an inter-rater reliability analysis was conducted. Two independent raters with anatomical training analyzed 20 thermal images from 20 professional football players (Canadian Football League), separate from the primary case subject. This cohort included players with and without injuries to ensure the protocol’s robustness across varied thermal patterns. Each rater, blinded to the other’s measurements, segmented the frontal knee and VMO for each image (40 total ROIs). For each ROI, maximum, average, and minimum temperature values were then extracted.

### 2.5. Agreement Between Manual and Automatic Segmentation

The agreement study used a repeated-measures design in which 50 thermal images were captured from a single participant over a four-month rehabilitation period. The subject was a 26-year-old male professional American football player (Defensive Back, Canadian Football League) who sustained a right lateral meniscus bucket-handle tear on 18 May 2023 and underwent arthroscopic meniscal repair on 6 July 2023. Thermal images were captured at multiple time points throughout the rehabilitation program to monitor healing progression and recovery milestones. Image selection was based solely on temporal spacing and thermal diversity.

Each image was segmented into two ROIs—the injured right frontal knee and the uninjured right VMO—yielding 100 data points for comparison. All images were segmented by the same rater, who completed manual segmentation of all 50 images using FLIR Thermal Studio Pro™ before proceeding to automatic segmentation using ThermoHuman^®^. To prevent measurement bias, the rater was blinded to ThermoHuman^®^ values during manual segmentation and to FLIR Thermal Studio Pro™ values during automatic segmentation. It is important to note that ThermoHuman^®^ does not offer a discrete VMO segment; its closest corresponding region is the lower vastus medialis. Our manual segmentation was therefore aligned with this broader region while focusing specifically on the VMO.

This longitudinal design ensured that a wide range of temperatures (27.9 °C to 37.2 °C) was included. If we had imaged 50 participants at a single time point, such as one day post-injury, the temperature range would likely have been much narrower. Using a range of data points over time was based on previous studies in agreement research, which suggest that repeated measures across a range of physiological states provide sufficient variability to assess consistency between measurement tools [29,30,31,32,33,34]. The temperature values in our dataset encompassed the expected thermal variability associated with early-phase inflammation (elevated temperature), mid-phase remodeling (reduced temperature), and later-stage reactivation (fluctuating increases during return to sport). By capturing images from both an injury site and a stable reference region across time, we obtained a sufficient range of values necessary for valid method comparison within this single-case feasibility illustration.

### 2.6. Statistical Analysis

All statistical analyses were performed using SPSS (Version 29.0, IBM Corp., Chicago, IL, USA). For both analyses, ICC values were interpreted according to the following thresholds: poor (<0.50), moderate (0.50–0.75), good (0.75–0.90), and excellent (>0.90) [35].

#### 2.6.1. Inter-Rater Reliability of Manual Segmentation

To evaluate the inter-rater reliability of manual segmentation, two-way random-effects, single-measurement, absolute agreement Intraclass Correlation Coefficients (ICC _(2,1)_) were calculated for each temperature metric (maximum, average, and minimum) across both right frontal knee and right VMO ROIs.

#### 2.6.2. Agreement Between Manual and Automatic Segmentation

To evaluate the agreement between manual and automatic segmentation, the same ICC _(2,1)_ model was applied for each temperature metric across both ROIs. Bland–Altman plots were also generated to visualize the magnitude of differences between methods and assess for systematic bias [29,36].

## 3. Results

### 3.1. Inter-Rater Reliability of Manual Segmentation

The manual segmentation protocol demonstrated good-to-excellent inter-rater reliability. As detailed in Table 1, reliability for the frontal knee was excellent across all temperature metrics, with ICC _(2,1)_ values of 0.991 for maximum, 0.995 for average, and 0.964 for minimum temperature readings. Reliability for the VMO was excellent for maximum (ICC _(2,1)_ = 0.967) and average (ICC _(2,1)_ = 0.981) temperature readings. The VMO minimum temperature was the exception, demonstrating good reliability (ICC _(2,1)_ = 0.779).

### 3.2. Agreement Between Manual and Automatic Segmentation

Excellent agreement was observed between the automatic ThermoHuman^®^ and the manual FLIR Thermal Studio Pro™ segmentation for all temperature measurements of the frontal knee and VMO. As shown in Table 2, all ICC values surpassed the threshold for excellent agreement (>0.90). For the frontal knee, ICCs were 0.967 for maximum, 0.949 for average, and 0.985 for minimum temperature. For the VMO, ICCs were 0.971 for maximum, 0.973 for average, and 0.980 for minimum temperature. All correlations were statistically significant (*p* < 0.001).

**Figure 6:**
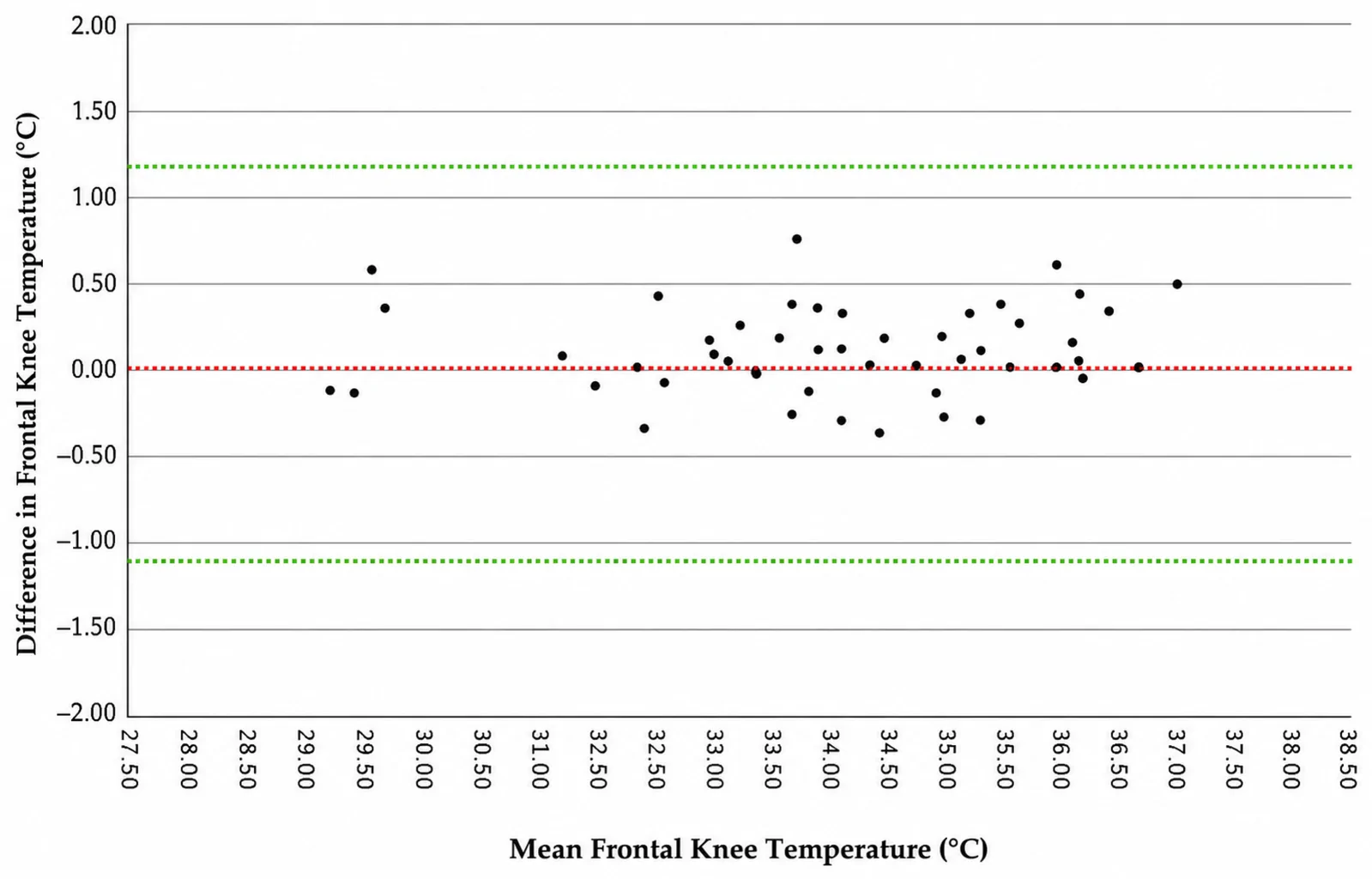
Bland–Altman plot showing the agreement of average right frontal knee temperature measurements between the manual and automatic segmentation methods.

**Figure 7:**
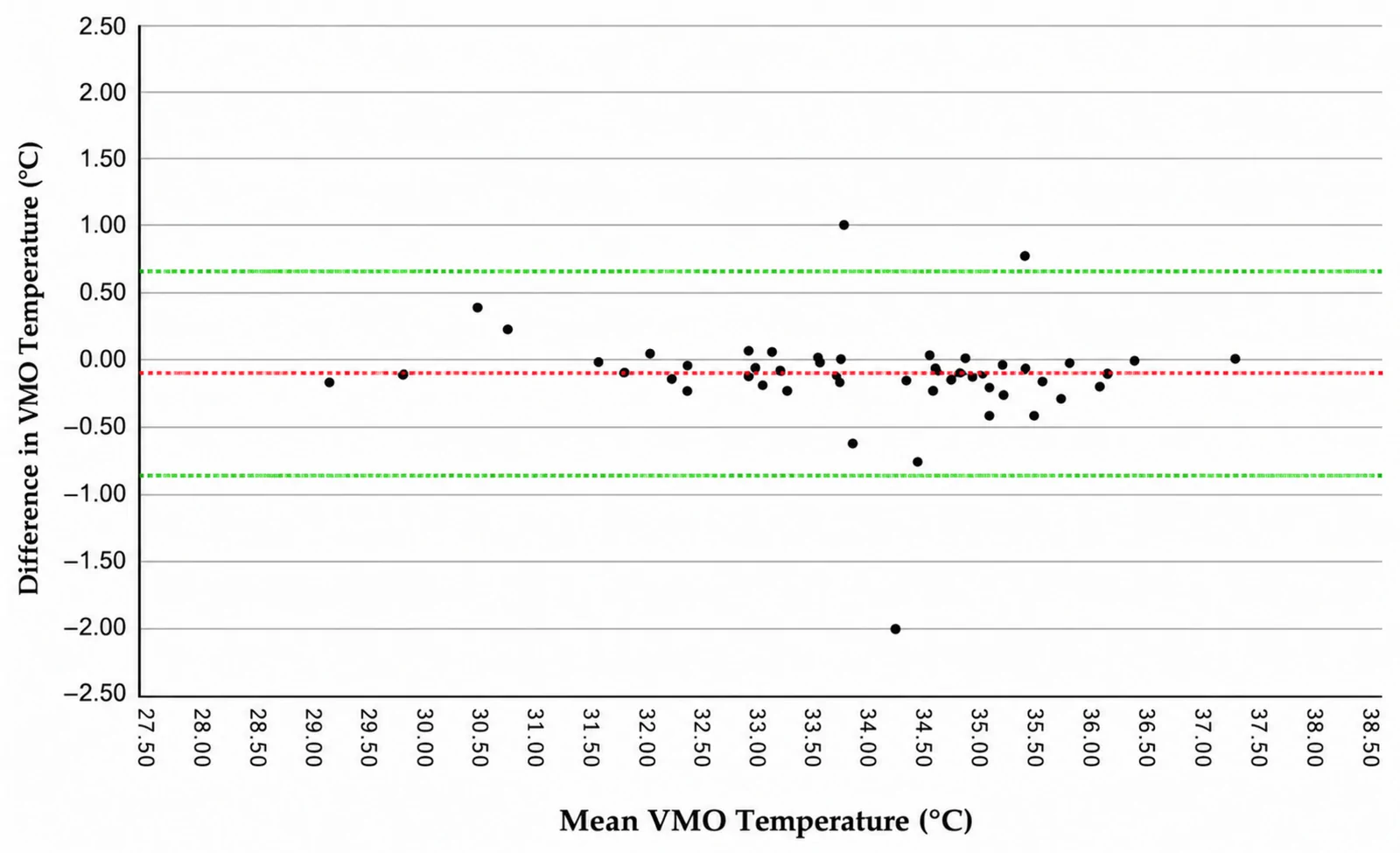
Bland–Altman plot showing the agreement of average right VMO temperature measurements between the manual and automatic segmentation methods.

The Bland–Altman analysis confirmed the high level of agreement. For the average frontal knee temperature (Figure 6), the mean difference was 0.039 °C, with 95% limits of agreement (LoA) from −1.091 °C to 1.168 °C. For the average VMO temperature (Figure 7), the mean difference was −0.128 °C, with narrower 95% LoA from −0.876 °C to 0.621 °C. In both plots, data points were evenly distributed around the mean, indicating no proportional bias.

## 4. Discussion

This study assessed the inter-rater reliability of the manual segmentation protocol, a crucial step for validating its use in clinical practice. The results showed excellent reliability for five of the six measurements, with only the VMO minimum temperature demonstrating good reliability (ICC = 0.779). This suggests that with a standardized, landmark-based protocol, different raters can achieve highly consistent results. As shown is the Appendix, the lower ICC for VMO minimum temperature is primarily attributable to a single outlier, where one rater’s segmentation in participant 1 inadvertently included cooler peripheral skin, leading to a 4.6 °C discrepancy between raters (Table A1). This temperature difference highlights IRT’s extreme sensitivity to ROI delineation, emphasizing that even minor overlap with the cooler skin barrier can significantly impact minimum temperature values. This finding underscores a critical practical consideration for clinicians: meticulous attention to ROI boundaries, especially when assessing minimum temperatures, is paramount to ensure reliable measurements. This outlier was retained to reflect real-world clinical conditions; excluding it would have resulted in excellent reliability across all VMO minimum measurements. This further underscores the importance of strict adherence to anatomical landmarks during segmentation. Overall, the high reliability observed across the cohort of 20 athletes suggests that the manual segmentation protocol is reproducible when applied with sound anatomical judgment, provided careful attention is paid to ROI definition, particularly for minimum temperature readings.

This study also demonstrated excellent agreement between automatic (ThermoHuman^®^) and manual (FLIR Thermal Studio Pro™) thermal segmentation methods for monitoring an injured knee and a functionally related muscle during rehabilitation. ICC values were excellent for all frontal knee measurements (0.967, 0.949, and 0.985 for maximum, average, and minimum temperature) and all VMO measurements (0.971, 0.973, and 0.980 for maximum, average, and minimum temperature), confirming that both segmentation approaches produce highly consistent temperature readings in this single-case feasibility illustration. These findings provide preliminary support for the use of the more accessible manual segmentation method for tracking thermal changes without compromising measurement consistency, though multi-subject replication is needed before broader conclusions can be drawn, thereby broadening the clinical applicability of IRT as a complementary monitoring tool.

It is important to contextualize the role of IRT within the broader landscape of rehabilitation monitoring. While IRT offers significant advantages in terms of non-invasiveness, accessibility, and the ability to provide frequent, real-time data on physiological changes, it is not intended to replace imaging modalities such as MRI. MRI remains indispensable for initial diagnosis, detailed anatomical assessment, and identifying structural pathologies that IRT cannot detect. Instead, IRT serves as a powerful complementary tool, providing dynamic physiological insights that enhance the overall monitoring strategy. For instance, while MRI might reveal the extent of a meniscal tear, IRT can track the inflammatory response and tissue healing progression over time, offering actionable data for rehabilitation adjustments. This complementary approach allows clinicians to leverage the strengths of each modality, providing a more comprehensive understanding of an athlete’s recovery and guiding return-to-play decisions with greater precision. Furthermore, it is crucial to recognize that persistent anatomical changes observed on MRI in asymptomatic individuals do not necessarily indicate ongoing pathology or risk of reinjury [10,11]. IRT, by focusing on physiological responses, can provide a more accurate reflection of the current tissue state and functional recovery, thereby avoiding misleading interpretations that might arise from relying solely on structural imaging in later stages of rehabilitation.

Our observed ICC values for inter-rater reliability are comparable to, and in some cases higher than, those observed in previous IRT reliability studies. The agreement ICCs, while similarly high in this single case, should be interpreted in the context of the single-subject feasibility design. In previous studies that measured reliability using IRT, ICCs of 0.865 were noted in patients with complex regional pain syndrome [14]. In addition, another study generated an ICC greater than 0.75 in chronic migraine evaluations [15]. The work of Molina-Payá et al. (2021), which examined the inter- and intra-examiner reliability of a new method for IRT analysis of the patellar tendon, showed very high reliability, with all lower limits of the ICC exceeding 0.84 [20]. Lastly, the study by Calvo-Lobo et al. concentrated on the reliability of the Manual Infrared Camera PCE-TC 30 for measuring skin temperature in athletes’ triceps surae muscles [17]. They found very high ICC values between 0.968 and 0.977, indicating the device’s consistent performance in capturing temperature readings [17]. Unlike our study, which assessed image segmentation reliability, their focus was primarily on the tool’s measurement reliability [17].

The high ICC values for the frontal knee may reflect localized heat concentration at the injury site, potentially related to inflammation and increased blood flow during healing [37,38,39]. This centralization likely ensures that both manual and automatic segmentation methods capture the same thermally active region. Future research directly measuring physiological parameters alongside IRT would help confirm these proposed mechanisms. Additionally, the FLIR E8 thermal camera is highly accurate in detecting temperature variations, so minor differences in region shape or boundaries between the two methods do not significantly affect the recorded maximum temperature, as long as the injury site is included within the segmented area. As a result, the likelihood of overlooking peak temperatures is low, and manual segmentation, despite being operator-dependent, aligns closely with automatic software like ThermoHuman^®^, which is designed to identify key anatomical regions.

Similarly, the excellent agreement observed for VMO temperature readings, especially for maximum values, can be explained by the muscle’s consistent functional role during rehabilitation. Although the VMO was not injured, it was selected due to its key role in knee stabilization and its targeted activation throughout rehabilitation. As the VMO is repeatedly engaged during exercises aimed at restoring knee function, the increased muscle activity results in localized heat concentration within this region. It is important to acknowledge the imperfect correspondence in VMO region delineation between the two methods: ThermoHuman^®^ encompassed a broader region (the entire vastus medialis muscle), potentially capturing more variation in temperature, while the manual method focused more precisely on the VMO muscle itself. Despite this anatomical discrepancy, the high ICC values indicate that both methods consistently measured the region of highest thermal activity within the muscle in this individual. This suggests that even with slight variations in ROI definition, both approaches may effectively capture the physiologically relevant thermal signature of the VMO, though this warrants confirmation in larger samples. These findings also demonstrate that both methods can produce highly comparable temperature measurements in uninjured but functionally involved muscles, supporting their potential clinical utility for monitoring rehabilitation progress.

The confidence intervals (CIs) observed in this study were consistently narrow across all temperature readings, further reinforcing the consistency of the agreement between manual and automatic methods within this single case. The smallest CI was observed for the minimum frontal knee temperature (ICC = 0.985; 95% CI: 0.965 to 0.993), while the widest CI occurred for the maximum frontal knee temperature (ICC = 0.967; 95% CI: 0.824 to 0.988). Across all six ICC calculations, the average 95% CI width was approximately 0.048. Narrow CIs suggest that the observed agreement is unlikely to be due to chance or sample-specific anomalies and that similar levels of agreement would likely be observed for this participant in comparable clinical settings. Such consistency is particularly important in rehabilitation contexts where small differences in thermal data can influence decisions about patient progress or treatment changes.

The Bland–Altman plots offer critical insight beyond correlation statistics by visualizing the agreement between the two measurement methods and identifying any systematic bias or variability [29,30]. In clinical settings, this type of analysis is essential because tools must show both high correlation (as provided by ICCs) and minimal measurement bias before they can be considered for interchangeable use [29,30]. For the frontal knee (Figure 6), the mean difference between ThermoHuman^®^ and manual measurements was 0.039 °C, with limits of agreement from −1.091 °C to +1.168 °C. Data points were evenly distributed around the mean, with no evidence of proportional bias, and all values fell within the limits of agreement, indicating excellent consistency across the full temperature range for this participant. The frontal knee likely showed this level of agreement due to centralized heat concentration from inflammation and tissue activity post-injury, which produced highly reproducible thermal patterns across both segmentation methods. For the VMO (Figure 7), the mean difference was slightly negative (−0.128 °C), and the limits of agreement were narrower (−0.876 °C to 0.621 °C), indicating not only high agreement but also less variability compared to the knee. Only three data points fell outside the limits for the VMO, indicating minor variability but overall reinforcing the observed consistency between methods in this case. The tighter clustering may be attributed to the muscle’s more uniform surface area and consistent recruitment during rehabilitation, which led to stable thermal outputs.

In addition to the strong statistical agreement, the raw temperature data presented in Table A2 of the Appendix further support the clinical promise of these findings. Across maximum, average, and minimum readings, the absolute temperature differences between manual and automatic methods were typically within 1 °C, with most varying by only a few tenths of a degree. While a small number of values exceeded this range, only one instance approached a 4 °C difference, representing an outlier rather than the norm. This clarification is important, as the typical magnitude of difference was low and well within clinically acceptable bounds. From a clinician’s perspective, such small discrepancies are unlikely to impact interpretation, especially when monitoring trends over time. Therefore, based on this feasibility illustration, both methods appear suitable to track rehabilitation progress in injured individuals across multiple sessions, without concern for meaningful deviation between tools.

## 5. Limitations

Several limitations should be considered when interpreting these findings. Foremost, both raters in the reliability analysis had formal anatomical training; variability might be higher among clinicians with less experience. Furthermore, the agreement analysis in this study is based on a longitudinal single-case design, utilizing 50 thermal images from one athlete over a four-month rehabilitation period. While this approach allowed for robust evaluation of agreement across a wide range of physiological states within a single individual, it inherently limits the generalizability of our findings to a broader population of injured athletes. The results of the agreement study should therefore be interpreted as a feasibility illustration of the segmentation methods, rather than a definitive statement on their applicability across diverse patient cohorts. Future research should aim to replicate these findings in multi-subject studies to assess broader generalizability. Additionally, the findings were limited to two specific anatomical regions of the lower extremity, and whether similar levels of agreement persist across other body segments remains to be confirmed. Moreover, the comparison for the VMO involved an inherent anatomical discrepancy, as ThermoHuman^®^ analyzes the broader lower vastus medialis region, whereas our manual protocol targeted the VMO specifically. While this imperfect correspondence was a limitation, the high agreement observed suggests both methods effectively captured the key thermal activity of the target muscle, indicating robustness despite differing ROI definitions. Also, all images were acquired with a single camera model under tightly controlled environmental conditions that may be difficult to replicate in typical clinical settings. Finally, variations in subcutaneous fat thickness (plicae) were not accounted for in this study, which is an important parameter that can influence surface temperature measurements and should be considered in future research.

## 6. Conclusions

This study provides two complementary contributions for clinicians working with IRT in musculoskeletal rehabilitation. First, the inter-rater reliability analysis, conducted across 20 professional athletes, confirms that manual thermal image segmentation using FLIR Thermal Studio Pro™ is highly reproducible between independent raters, supporting its use as a practical and consistent tool. Second, as a longitudinal single-case feasibility illustration, this study demonstrates excellent agreement between manual segmentation and automatic segmentation using ThermoHuman^®^, suggesting that clinicians can obtain comparable temperature measurements using accessible manual software alternatives. The generalizability of the agreement findings is inherently limited by the single-subject design, and future research should aim to replicate these results across diverse injuries, anatomical regions, and multi-subject cohorts.

## Figures and Tables

**Table 1:** Inter-rater reliability (ICC _(2,1)_) for manual segmentation of the right frontal knee and VMO.

Anatomical Region	Temperature Reading	ICC _(2,1)_	[95% CI]	*p*-Value
Frontal Knee	Maximum	0.991 *	[0.978, 0.996]	*p* < 0.001
Frontal Knee	Average	0.995 *	[0.987, 0.998]	*p* < 0.001
Frontal Knee	Minimum	0.964 *	[0.911, 0.985]	*p* < 0.001
VMO	Maximum	0.967 *	[0.919, 0.987]	*p* < 0.001
VMO	Average	0.981 *	[0.954, 0.993]	*p* < 0.001
VMO	Minimum	0.779 *	[0.530, 0.905]	*p* < 0.001

*: Statistically significant (*p* < 0.001).

**Table 2:** Agreement (ICC _(2,1)_) between manual and automatic segmentation methods for temperature readings of the right frontal knee and VMO.

Anatomical Region	Temperature Reading	ICC _(2,1)_	[95% CI]	*p*-Value
Frontal Knee	Maximum	0.967 *	[0.824, 0.988]	*p* < 0.001
Frontal Knee	Average	0.949 *	[0.912, 0.971]	*p* < 0.001
Frontal Knee	Minimum	0.985 *	[0.965, 0.993]	*p* < 0.001
VMO	Maximum	0.971 *	[0.913, 0.987]	*p* < 0.001
VMO	Average	0.973 *	[0.951, 0.985]	*p* < 0.001
VMO	Minimum	0.980 *	[0.966, 0.989]	*p* < 0.001

*: Statistically significant (*p* < 0.001).

## Data Availability

The original contributions presented in this study are included in the article; further inquiries can be directed to the corresponding author.

## Appendix A

**Table A1:** Inter-rater reliability raw data: right frontal knee and VMO maximum, minimum, and average temperature readings as measured with FLIR Thermal Studio Pro™.

Image ID	Rater 1 Frontal Knee Max. (°C)	Rater 2 Frontal Knee Max. (°C)	Rater 1 Frontal Knee Min. (°C)	Rater 2 Frontal Knee Min. (°C)	Rater 1 Frontal Knee Avg. (°C)	Rater 2 Frontal Knee Avg. (°C)	Rater 1 VMO Max. (°C)	Rater 2 VMO Max. (°C)	Rater 1 VMO Min. (°C)	Rater 2 VMO Min. (°C)	Rater 1 VMO Avg. (°C)	Rater 2 VMO Avg. (°C)
1	29.9	29.8	26.9	26.9	28.1	28	30	30	28.3	23.7	29.3	29.1
2	32.4	32.3	28.5	28.5	30.4	30.2	32.1	32.1	28.8	28	31.6	31.1
3	29.7	29.7	26.4	26.4	28	27.9	29.7	29.7	28.1	28.4	29.2	29.3
4	32.3	32.3	29.8	29.6	31	30.8	31.6	31.9	30.4	30.6	31.2	31.3
5	30.5	30.4	27.1	27	28.5	28.3	29.4	29.8	27.5	27.6	28.8	29.1
6	30.1	30.1	27.1	27.3	28.5	28.6	30	29	27.2	27.8	28.6	28.6
7	31.8	31.8	28.2	28.6	29.6	29.9	31.1	30.8	29.3	29.5	30.3	30.5
8	31.7	31.9	29.1	29.1	30.1	30.2	31.2	31.4	29.8	30.1	31	31
9	29.7	29.7	26.3	26.3	27.7	27.7	29	29	27.5	27.4	28.4	28.2
10	31.2	30.8	26.1	26.1	28.2	28.2	30.8	31.3	28.3	27.9	29.4	29.9
11	30.1	30.1	26.6	26.6	28.1	28.1	30.1	29.8	28.6	27.9	29.4	28.9
12	32.6	32.6	29.9	29.3	31.3	31.3	31.2	30.9	30.2	30	30.7	30.6
13	29.5	29.4	26.9	26.9	28.1	28.1	29.9	29.9	28.9	28.5	29.5	29.2
14	33.5	33.5	30.1	30.1	31.5	31.5	32.6	32.8	31.6	31.6	32.1	32.2
15	33.8	34.4	30	30	32.2	32.6	34.8	34.7	31.9	31.9	33.5	33.3
16	32.9	32.9	30.1	30.3	31.4	31.4	31.8	31.8	30.3	30.5	31	30.9
17	31.9	31.9	29.4	27.9	30.5	30.5	31.9	31.1	29.7	30.1	30.8	30.6
18	30.8	30.6	28	28.4	29.4	29.5	30.2	30.3	28.3	28.2	29.4	29.7
19	31.6	31.7	28.7	28.7	30.1	30.2	31.8	31.9	30.4	30.2	31	30.9
20	32.2	32.3	30	30	30.9	30.9	32.2	32.2	31.1	30.5	31.7	31.6

**Table A2:** Agreement raw data: right frontal knee and VMO maximum, minimum, and average temperature readings as measured with FLIR Thermal Studio Pro™ and ThermoHuman®.

Image ID	Image Capture Date	Manual Knee Max. (°C)	Automatic Knee Max. (°C)	Manual Knee Min. (°C)	Automatic Knee Min. (°C)	Manual Knee Avg. (°C)	Automatic Knee Avg. (°C)	Manual VMO Max. (°C)	Automatic VMO Max. (°C)	Manual VMO Min. (°C)	Automatic VMO Min. (°C)	Manual VMO Avg. (°C)	Automatic VMO Avg. (°C)
1	Pre-Season	30.4	30.4	28.2	28.7	29.4	29.5	31.7	31.4	29.4	29.0	30.7	30.3
2	Pre-Surgery	30.8	30.9	28.5	28.8	29.6	29.7	32.0	32.1	29.1	29.1	30.9	30.7
3	7 July 2023	33.4	33.1	30.3	31.4	32.0	32.3	33.4	33.4	30.1	31.8	32.3	32.5
4	9 July 2023	37.0	36.6	34.9	34.9	35.9	35.7	36.5	37.0	35.5	35.4	36.0	36.2
5	10 July 2023	32.3	32.1	28.3	28.6	31.7	31.8	32.6	32.8	30.1	31.0	32.1	32.1
6	11 July 2023	33.8	33.7	32.3	32.3	33.1	33.1	33.4	33.7	32.0	32.1	33.0	33.0
7	12 July 2023	32.4	31.9	30.1	30.5	31.5	31.4	32.2	32.4	30.3	30.4	31.6	31.6
8	13 July 2023	33.8	33.7	31.8	31.8	33.1	32.8	33.7	33.7	32.2	32.0	33.2	33.2
9	14 July 2023	37.8	37.4	35.7	35.7	37.0	36.5	37.9	37.9	35.4	35.9	37.3	37.3
10	16 July 2023	31.1	30.8	28.6	28.3	30.1	29.5	29.8	30.9	28.3	28.3	29.1	29.3
11	18 July 2023	34.9	32.8	31.0	32.4	33.9	33.8	34.8	34.9	31.5	31.7	34.3	34.5
12	19 July 2023	34.1	33.8	31.6	31.6	32.9	32.8	33.5	34.1	32.1	32.1	32.9	33.0
13	21 July 2023	35.8	35.7	33.5	33.7	35.1	34.8	35.7	36.2	33.3	33.6	34.9	35.3
14	25 July 2023	35.2	34.9	33.2	33.3	34.3	34.1	35.1	35.2	32.9	32.9	34.3	33.3
15	26 July 2023	36.9	36.2	34.7	34.7	36.0	35.4	36.6	36.9	34.6	34.7	36.1	36.2
16	27 July 2023	37.2	36.6	35.0	35.0	36.3	36.0	37.1	37.2	35.1	35.2	36.4	36.4
17	28 July 2023	35.7	35.3	33.5	33.6	34.8	34.6	35.4	35.7	33.8	33.5	34.8	34.9
18	31 July 2023	34.0	33.2	31.0	31.1	32.5	32.1	34.0	34.1	32.7	32.7	33.6	33.6
19	1 August 2023	34.7	34.3	32.2	32.1	33.6	33.2	34.6	34.7	32.5	32.5	33.7	33.9
20	3 August 2023	34.6	34.2	32.1	32.0	33.4	33.2	34.4	34.6	32.5	32.4	33.2	33.3
21	5 August 2023	35.7	35.3	33.2	33.4	34.0	34.3	35.7	35.6	32.9	33.5	34.6	34.6
22	6 August 2023	35.2	34.6	32.4	32.8	34.0	33.7	35.2	35.2	34.0	33.9	34.6	34.7
23	8 August 2023	34.7	34.3	32.3	32.3	33.8	33.5	34.6	34.7	32.7	32.7	33.8	33.8
24	9 August 2023	36.3	35.9	34.3	34.3	35.5	35.2	36.0	36.3	33.9	33.8	35.4	35.5
25	12 August 2023	36.3	35.9	34.0	34.1	35.4	35.0	36.0	36.1	33.9	33.7	35.2	35.3
26	16 August 2023	37.0	36.6	34.6	34.6	35.9	35.8	36.1	37.0	34.8	34.8	35.5	35.7
27	17 August 2023	35.1	34.9	33.0	33.0	34.1	34.1	34.9	35.0	32.3	32.3	33.6	33.6
28	19 August 2023	34.6	34.6	32.7	32.5	33.5	33.6	34.6	36.2	32.6	33.6	33.3	35.3
29	20 August 2023	34.4	34.3	32.6	32.6	33.7	33.6	34.4	34.5	33.2	33.2	33.7	33.8
30	22 August 2023	36.5	36.3	34.8	34.8	35.7	35.7	36.3	36.5	34.8	34.7	35.8	35.8
31	26 August 2023	30.9	30.8	28.8	28.8	30.1	29.8	30.6	30.9	27.9	28.0	29.8	29.9
32	29 August 2023	36.7	36.7	34.8	34.9	35.9	35.9	36.6	36.6	34.7	34.8	35.1	35.4
33	30 August 2023	35.5	35.3	34.0	34.1	34.9	34.8	35.4	35.5	34.3	34.1	34.9	34.9
34	31 August 2023	36.1	35.9	34.4	34.4	35.3	35.3	35.6	35.9	34.3	34.3	34.9	35.0
35	5 September 2023	37.0	36.9	35.4	35.6	36.4	36.4	36.8	37.0	34.5	34.3	35.6	35.9
36	6 September 2023	33.8	33.9	32.3	32.3	33.1	33.1	33.9	33.9	32.3	32.2	33.0	33.2
37	7 September 2023	36.8	36.4	34.8	34.9	36.1	35.7	36.6	36.8	33.8	33.8	35.3	35.7
38	20 September 2023	35.5	35.5	33.7	34.0	34.6	34.7	35.5	35.3	33.6	33.4	34.5	34.7
39	21 September 2023	35.8	35.6	34.2	34.2	35.1	35.0	35.7	35.8	34.3	34.4	35.0	35.1
40	26 September 2023	33.0	32.8	31.2	31.3	32.1	32.1	32.7	32.8	31.2	31.2	31.8	31.9
41	30 September 2023	36.5	35.9	34.2	34.4	35.4	35.3	36.1	35.9	34.5	34.3	35.8	35.0
42	1 October 2023	35.3	35.1	33.8	33.9	34.5	34.5	35.0	35.5	33.7	33.6	34.6	34.7
43	2 October 2023	33.8	33.4	31.7	31.7	32.8	32.6	33.7	33.7	31.9	31.9	33.0	33.1
44	4 October 2023	35.9	35.9	33.3	33.8	34.6	34.9	36.0	35.8	33.6	33.5	34.7	34.9
45	11 October 2023	33.2	33.1	31.4	31.6	32.3	32.4	33.0	33.4	31.3	31.2	32.2	32.4
46	18 October 2023	34.8	35.0	32.4	32.7	33.7	34.0	35.0	35.8	33.2	33.5	34.1	34.9
47	19 October 2023	34.6	34.4	32.5	33.1	33.8	33.1	34.4	35.0	33.2	33.3	33.6	34.2
48	31 October 2023	34.3	34.4	32.4	32.5	33.3	33.5	34.4	34.4	32.2	32.0	33.2	33.4
49	7 November 2023	33.9	33.1	31.7	31.9	32.8	32.7	33.6	33.7	31.5	31.5	32.4	32.5
50	21 November 2023	36.1	35.9	33.8	34.4	34.9	35.2	36.0	36.4	34.4	34.3	35.0	35.2

## References

[1] Castonguay T., Dover G. (2023). Infrared Thermography—A Novel Tool for Monitoring Fracture Healing: A Critically Appraised Topic with Evidence-Based Recommendations for Clinical Practice. J. Sport Rehabil..

[2] Reed C.L., Saatchi R., Ramlakhan S. Infrared thermal imaging for bone fracture identification and monitoring of fracture healing: A review of the latest developments. Proceedings of the Seventeenth International Conference on Condition Mon-itoring and Asset Management.

[3] der Strasse W.A., Campos D.P., Mendonça C.J.A., Soni J.F., Mendes J., Nohama P. (2021). Evaluation of Tibia Bone Healing by Infrared Thermography: A Case Study. J. Multidiscip. Healthc..

[4] der Strasse W.A., Campos D.P., Mendonça C.J.A., Soni J.F., Tuon F., Mendes J., Nohama P. (2022). Evaluating physiological progression of chronic tibial osteomyelitis using infrared thermography. Res. Biomed. Eng..

[5] Usamentiaga R., Venegas P., Guerediaga J., Vega L., Molleda J., Bulnes F.G. (2014). Infrared Thermography for Temperature Measurement and Non-Destructive Testing. Sensors.

[6] Ćurković S., Antabak A., Halužan D., Luetić T., Prlić I., Šiško J. (2015). Medical thermography (digital infrared thermal imaging – DITI) in paediatric forearm fractures—A pilot study. Injury.

[7] Wessman B.V., Moriarity A.K., Ametlli V., Kastan D.J. (2014). Reducing Barriers to Timely MR Imaging Scheduling. RadioGraphics.

[8] Cournane S., Conway R., Creagh D., Byrne D., Sheehy N., Silke B. (2016). Radiology imaging delays as independent predictors of length of hospital stay for emergency medical admissions. Clin. Radiol..

[9] Canadian Association of Radiologists (2019). Value of Radiology Part II—Excessive Wait Times for Medical Imaging Hurts Canadians in More Ways than One. https://car.ca/wp-content/uploads/2025/03/value-of-radiology-part-2-en.pdf.

[10] Malliaropoulos N., Isinkaye T., Tsitas K., Maffulli N. (2010). Reinjury After Acute Posterior Thigh Muscle Injuries in Elite Track and Field Athletes. Am. J. Sports Med..

[11] Martin R.L., Cibulka M.T., A Bolgla L., A Koc T., Loudon J.K., Manske R.C., Weiss L., Christoforetti J.J., Heiderscheit B.C. (2022). Hamstring Strain Injury in Athletes. J. Orthop. Sports Phys. Ther..

[12] Majano C., Garcia-Unanue J., Fernández-Cuevas I., Escamilla-Galindo V., Alonso-Callejo A., Sanchez-Sanchez J., Gallardo L., Felipe J.L. (2023). Association between physical demands, skin temperature and wellbeing status in elite football players. Sci. Rep..

[13] Ahmed I., Ishtiaq S. (2021). Reliability and Validity: Importance in medical research. J. Pak. Med. Assoc..

[14] Choi E., Lee P., Nahm F.S. (2013). Interexaminer reliability of infrared thermography for the diagnosis of complex regional pain syndrome. Ski. Res. Technol..

[15] Antonaci F., Rossi E., Voiticovschi-Iosob C., Volta G.D., Marceglia S. (2018). Frontal infrared thermography in healthy individuals and chronic migraine patients: Reliability of the method. Cephalalgia.

[16] Rossignoli I., Benito P.J., Herrero A.J. (2014). Reliability of infrared thermography in skin temperature evaluation of wheelchair users. Spinal Cord.

[17] Calvo-Lobo C., San-Antolín M., García-García D., Becerro-De-Bengoa-Vallejo R., Losa-Iglesias M.E., Cosín-Matamoros J., Casado-Hernández I., Martínez-Jiménez E.M., Mazoteras-Pardo V., Rodríguez-Sanz D. (2023). Intra- and inter-session reliability and repeatability of an infrared thermography device designed for materials to measure skin temperature of the triceps surae muscle tissue of athletes. PeerJ.

[18] James C., Richardson A., Watt P., Maxwell N. (2014). Reliability and validity of skin temperature measurement by telemetry thermistors and a thermal camera during exercise in the heat. J. Therm. Biol..

[19] Requena-Bueno L., Priego-Quesada J.I., Jimenez-Perez I., Gil-Calvo M., Pérez-Soriano P. (2020). Validation of ThermoHuman automatic thermographic software for assessing foot temperature before and after running. J. Therm. Biol..

[20] Molina-Payá J., Ríos-Díaz J., Martínez-Payá J.J. (2019). Inter and intraexaminer reliability of a new method of infrared thermography analysis of patellar tendon. Quant. Infrared Thermogr. J..

[21] ThermoHuman (2024). ThermoHuman Thermal Image Protocols: Description.

[22] Pakarinen T., Joutsen A., Oksala N., Vehkaoja A. (2023). Assessment of chronic limb threatening ischemia using thermal imaging. J. Therm. Biol..

[23] Systems F.L.I.R. FLIR Ex Series User Manual. https://www.instrumart.com/assets/FLIR-Ex-Series-Manual.pdf?srsltid=AfmBOoq5qJyqcaqHtQFg45HlQdYWzjL….

[24] Ammer K. (2008). The Glamorgan Protocol for recording and evaluation of thermal images of the human body. Thermol. Int..

[25] Moreira D.G., Costello J.T., Brito C.J., Adamczyk J.G., Ammer K., Bach A.J., Costa C.M., Eglin C., Fernandes A.A., Fernández-Cuevas I. (2017). Thermographic imaging in sports and exercise medicine: A Delphi study and consensus statement on the measurement of human skin temperature. J. Therm. Biol..

[26] American Academy of Thermology (2024). Guidelines for Neuro-Musculoskeletal Infrared Medical Thermology & Sympathetic Skin Response Studies.

[27] Carvajal-Fernández O., Álvarez-Prats D., Medina-Mirapeix F., Minaya-Muñoz F. (2016). Effects of percutaneous needle electrolysis of the patellar tendon on local and contralateral temperature measured with infrared thermography. Rev. Fisioter. Invasiva J. Invasive Tech. Phys. Ther..

[28] Pokora I., Drzazga Z., Wyderka P., Binek M. (2023). Determination of the Effects of a Series of Ten Whole-Body Cryostimulation Sessions on Physiological Responses to Exercise and Skin Temperature Behavior following Exercise in Elite Athletes. J. Clin. Med..

[29] Bland J.M., Altman D.G. (1986). Statistical methods for assessing agreement between two methods of clinical measurement. Lancet.

[30] Bland J.M., Altman D.G. (1999). Measuring agreement in method comparison studies. Stat. Methods Med. Res..

[31] Shrout P.E., Fleiss J.L. (1979). Intraclass correlations: Uses in assessing rater reliability. Psychol. Bull..

[32] Watson P., Petrie A. (2010). Method agreement analysis: A review of correct methodology. Theriogenology.

[33] Donner A., Eliasziw M. (1987). Sample size requirements for reliability studies. Stat. Med..

[34] Giraudeau B., Mary J.Y. (2001). Planning a reproducibility study: How many subjects and how many replicates per subject for an expected width of the 95 per cent confidence interval of the intraclass correlation coefficient. Stat. Med..

[35] Cocchetti D.V. (1999). Sample Size Requirements for Increasing the Precision of Reliability Estimates: Problems and Proposed Solutions. J. Clin. Exp. Neuropsychol..

[36] Rankin G., Stokes M. (1998). Reliability of assessment tools in rehabilitation: An illustration of appropriate statistical analyses. Clin. Rehabil..

[37] Malanga G.A., Yan N., Stark J. (2014). Mechanisms and efficacy of heat and cold therapies for musculoskeletal injury. Postgrad. Med..

[38] De Marziani L., Boffa A., Angelelli L., Andriolo L., Di Martino A., Zaffagnini S., Filardo G. (2023). Infrared Thermography in Symptomatic Knee Osteoarthritis: Joint Temperature Differs Based on Patient and Pain Characteristics. J. Clin. Med..

[39] Spataro M., Crisafulli D., Marchis C.D., Risitano G., Milone D. (2025). Effect of Weight Distribution on Knee Joint Temperature Pattern Under Fatigue Condition. Eng. Proc..

